# AIDS-related Kaposi sarcoma of the oral cavity

**DOI:** 10.1590/0037-8682-0133-2023

**Published:** 2023-06-02

**Authors:** Allan Vinícius Martins-de-Barros, Marianne de Vasconcelos Carvalho, Fábio Andrey da Costa Araújo

**Affiliations:** 1 Universidade de Pernambuco, Faculdade de Odontologia de Pernambuco, Programa de Pós-Graduação em Odontologia, Recife, PE, Brasil.; 2 Hospital Universitário Oswaldo Cruz, Centro Integrado de Anatomia Patológica, Recife, PE, Brasil.; 3 Hospital Universitário Oswaldo Cruz, Departamento de Cirurgia e Traumatologia Bucomaxilofacial, Recife, PE, Brasil.

A 25-year-old man with recently diagnosed acquired immunodeficiency syndrome (AIDS) was referred for consultation because of multiple intraoral lesions that had developed within past 2 months. Physical examination revealed diffuse multinodular purplish-red lesions with spontaneous bleeding affecting both the maxillary and mandibular gingiva (Figure 1). Histopathology of an incisional biopsy revealed a fascicular arrangement of spindle cells with congested blood vessels and hemorrhage in a telangiectatic pattern (Figure 2A). Immunohistochemistry for human herpesvirus 8 (HHV-8) was diffusely positive (Figure 2B), confirming the diagnosis of Kaposi sarcoma (KS). Complete remission of the oral lesions was achieved after 16 months of antiretroviral therapy (ART) and liposomal doxorubicin chemotherapy. No signs of relapse were observed at 1 year post-treatment follow-up (Figure 3).

**FIGURE 1:**
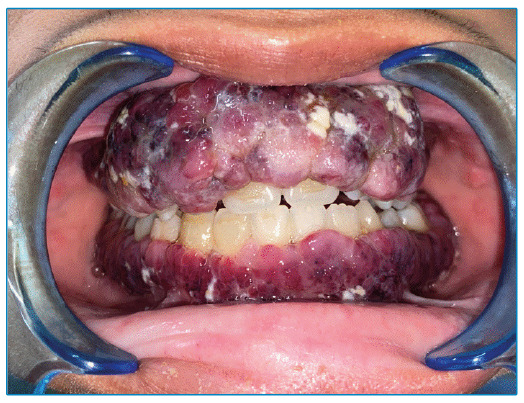
Initial clinical presentation showing multinodular lesions of the maxillary and mandibular gingiva.

**FIGURE 2:**
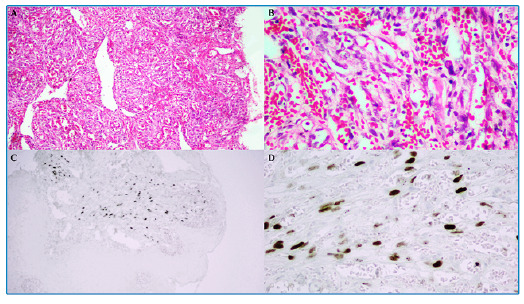
Histopathology of an incisional biopsy of an oral lesion. **A,** Photomicrography showing a fascicular arrangement of spindle cells with many congested blood vessels and hemorrhage in a telangiectatic pattern (hematoxylin and eosin stain; original magnification ×100) **B,** Higher magnification (hematoxylin and eosin stain; original magnification ×400). **C,** Photomicrography showing diffuse nuclear immunoreactivity for human herpesvirus 8 (HHV-8) in neoplastic spindle cells (DAB stain; original magnification ×100). D, Higher magnification of nuclear immunoreactivity for HHV-8 (DAB stain; original magnification ×400).

**FIGURE 3:**
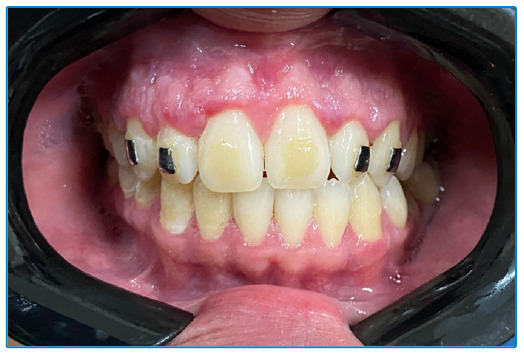
The gingiva 1 year after completing treatment.

KS is a multifocal angioproliferative disorder associated with HHV-8^1^. AIDS-related KS, the most common and aggressive variant, is the most frequent malignancy affecting people living with HIV^2^, in whom it often presents with mucocutaneous and/or visceral involvement. The oral cavity is affected in up to 70% of cases, and oral KS is sometimes the first manifestation of undiagnosed HIV infection^1^. According to the AIDS Clinical Trials Group, extensive oral involvement is a sign of advanced KS that worsens survival^3^. Although ART plays an important role in KS prevention and improves survival, factors such as delayed diagnosis, suboptimal linkage to care, and limited access to effective chemotherapy regimens hinder the management of advanced KS, especially in low- and middle-income countries^2^ ^,^ ^3^.

## ETHICS

All procedures involving human participants were in accordance with the ethical standards of the institutional and/or national research committee and with the 1964 Helsinki Declaration and its later amendments or comparable ethical standards.
